# The major toxins of Bacillus cereus – Current knowledge on distribution, regulation and mechanism of action

**DOI:** 10.17179/excli2026-9314

**Published:** 2026-04-17

**Authors:** Manar Albahri, Nadja Jessberger

**Affiliations:** 1 Institute of Food Quality and Food Safety, University of Veterinary Medicine Hannover, Bischofsholer Damm 15, 30173, Hanover, Germany

**Keywords:** food infection, food intoxication, haemolysin BL, non-haemolytic enterotoxin, cytotoxin K, cereulide

## Abstract

The ubiquitous soil bacterium *Bacillus cereus* is one of the major pathogens causing toxin-based foodborne diseases, manifesting as diarrhoeal or emetic syndrome. Main symptoms of the former are diarrhoea and abdominal pain, caused by the proteinaceous enterotoxins haemolysin BL (Hbl), non-haemolytic enterotoxin (Nhe) and cytotoxin K (CytK). Responsible for the latter is the cyclic dodecadepsipeptide cereulide, leading to nausea and vomiting, and, in severe and rare cases, also to organ failure and death. This review first addresses the complex taxonomy of the *B. cereus* group as well as the distribution of toxin genes within this group. Further, recent developments in studying the multifaceted intrinsic and extrinsic factors regulating enterotoxin and *ces* gene expression and toxin secretion are summarised. Special emphasis is placed on the latest findings regarding the mode of action of the pore-forming toxins, as well as on specific target structures and receptors, and the activation of cellular signalling pathways and inflammatory/apoptotic responses upon toxin exposure. Production and toxicity of the relatively new discovered cereulide isoforms are also discussed. A profound understanding of the fundamental processes of toxin formation and action is essential for accurate risk assessment of *B. cereus* isolates and for improving diagnostic procedures to increase food safety.

See also the graphical abstract(Fig. 1).

## Introduction

**Figure 2:**
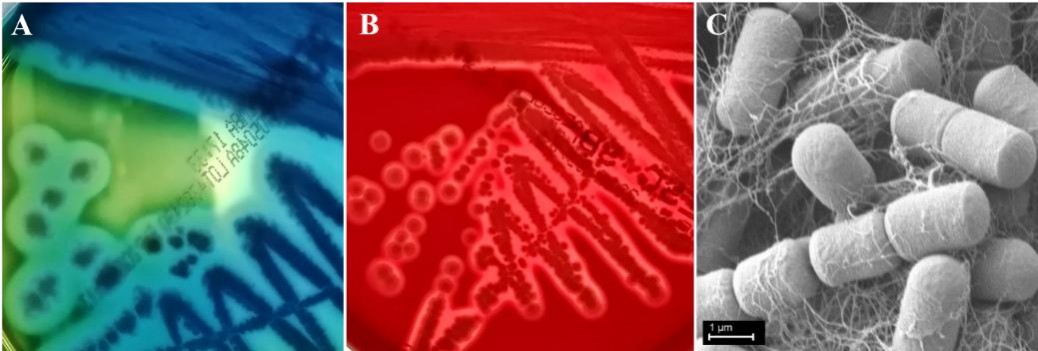
Characteristics of *B. cereus*: (A) Blue appearance on PEMBA (polymyxin pyruvate egg yolk mannitol bromothymol blue agar) due to lack of mannite cleavage and protein degradation. Opaque zones around the colonies due to lecithinase activity. (B) Growth on sheep blood agar, here with complete haemolysis. (C) Electron microscope image. Images: N. Jessberger

*Bacillus cereus* (Figure 2(Fig. 2)) is a spore-forming, Gram positive, and facultative anaerobic bacterium that was first isolated and described from an air sample by Frankland and Frankland (1887[77]). In a study from 1906, which was preceded by food poisoning after the consumption of Königsberger meatballs, it was initially characterised as* Bacillus peptonificans* (Lubenau, 1906[147]). Its typical waxy colony morphology, strong haemolysis, ability to liquefy gelatine, rapid sporulation, etc. provided initial evidence of its role as a pathogen in gastrointestinal diseases. This was confirmed in 1955, after numerous people in a hospital in Norway developed diarrhoea after eating vanilla sauce (Hauge, 1955[96]).

The bacterium is widespread in the environment, especially in soil. This is why it can be found in a variety of different foods (Johnson, 1984[119]; Kramer and Gilbert, 1989[129]). Once introduced into a food processing plant, the formation of biofilms plays an important role, which makes the bacterium resistant towards cleaning and disinfection measures (Anderson Borge et al., 2001[6]; Nam et al., 2014[173]; Ryu and Beuchat, 2005[198]). Even more challenging are the highly resistant spores, which are often unaffected by heat, low pH, radiation, dryness or other food preservation strategies (Carlin, 2011[33]; Jessberger et al., 2020[110]; Nicholson et al., 2000[179]; Setlow, 2006[207], 2014[206]; Stenfors Arnesen et al., 2008[214]). As soon as the spores come into contact with organic material in the soil, in food or in the host's gastrointestinal tract, germination is initiated by signalling the availability of nutrients (Abee et al., 2011[1]; Clavel et al., 2004[46]; Wijnands et al., 2006[233], 2007[232]). If this occurs in processed food, there is hardly any competing accompanying flora.

In addition to its role as a spoilage organism (Andersson et al., 1995[7]; De Jonghe et al., 2010[50]; Hendrickx et al., 2012[102]; Pepe et al., 2003[185]), *B. cereus* is primarily responsible for two types of gastrointestinal diseases. The first type, whose main symptoms are watery diarrhoea and abdominal pain, is a foodborne infection. The first symptoms appear after approx. eight to 16 hours. This incubation period is typical for a 'toxico-infection' in which the enterotoxins are produced by vegetative *B. cereus* in the small intestine (Dietrich et al., 2021[56]; Jessberger et al., 2020[110]; Logan, 2012[146]; Stenfors Arnesen et al., 2008[214]). Enterotoxins already present in foods are not significant, as they are sensitive towards heat, acid and proteolytic degradation (Ceuppens et al., 2012[39]). The course of the disease is usually self-limiting after approx. 12 to 24 hours. The infectious dose is approx. 10^5^-10^8^ CFU/g of food, although cases with higher and lower doses have also been described (Dietrich et al., 2021[56]; Jessberger et al., 2020[110]; Logan, 2012[146]; Stenfors Arnesen et al., 2008[214]). In particular with regard to the resistant spores, a much smaller number is likely to be sufficient to cause disease (Clavel et al., 2004[46]). The second, emetic form of the disease is characterised by the main symptoms of vomiting and nausea, which can occur as early as half an hour after consuming the contaminated food. This is a classic case of food intoxication, in which the highly resistant toxin cereulide is already present in the contaminated food and the bacteria themselves do not need to be ingested (Agata et al., 1994[3]). However, it is estimated that approx. 10^3^-10^5^ CFU/g of food is required to produce sufficiently high levels of toxin to cause illness (Agata et al., 1994[3]; Dietrich et al., 2021[56]; Jessberger et al., 2020[110]; Stenfors Arnesen et al., 2008[214]).

Statistics indicate that *B. cereus* is responsible for 1.4-12 % of global foodborne outbreaks, which is likely to be an underestimate (Grutsch et al., 2018[90]). For the year 2024, the European Food Safety Authority (EFSA) registered 127 foodborne outbreaks caused by *B. cereus* toxins, which corresponds to 1.9 % of reported outbreaks, including 3,315 cases of illness, 49 hospitalisations and nine deaths, which is the so far highest number reported for this agent (EFSA and ECDC, 2025[69]). Emetic and diarrhoeal food poisoning outbreaks associated with *B. cereus *are summarised in earlier review articles (from 1950 to 2019: Jessberger et al., 2020[110]; from 1984 to 2019: Leong et al., 2023[133]). More recent reports include a foodborne outbreak in a kindergarten in Korea, where *nhe*-positive *B. cereus* were detected in rectal and environmental (food) samples (Kim et al., 2023[124]), a foodborne outbreak in two middle schools in Chongqing, China, where students mainly suffered from vomiting after consuming contaminated rice noodles (Li et al., 2023[138]), and a combined outbreak (*B. cereus* and *Clostridium perfringens*) among hospital workers in Alaska, who mainly suffered from diarrhoea or abdominal cramping after consumption of contaminated ham and pulled pork sandwiches (Newell et al., 2024[177]). Even where (molecular biological) detection methods are not yet widely used, awareness of the risk posed by *B. cereus* is increasing, enabling outbreak investigations and/or tracing of contamination sources, as in the case of acute gastroenteritis in hospitalised children in Nigeria (David et al., 2024[49]) or in a food poisoning outbreak in a secondary school in Uganda, where students suffered from abdominal pain and diarrhoea following consumption of a cornmeal and beans (Namara et al., 2025[174]). More severe cases, including organ failure and death, are continuing to be caused by emetic *B. cereus*. Reports range from severe liver dysfunction in a female adult after eating a contaminated rice salad in Switzerland (Chatelanat et al., 2024[40]) as well as in five immunocompetent adults after consuming fried rice balls contaminated with extraordinary high levels of *B. cereus* and cereulide in Austria (Schreiber et al., 2021[204]), and multi-organ failure in an 11-year old girl in France resulting in hepatic transplantation (Thery et al., 2022[218]), to multi-organ failure resulting in mortality of an immunocompetent 40-year-old woman after consuming a reheated fried rice meal in Australia (Colaco et al., 2021[47]). Unusually, in a study from 2023, various *Bacillus thuringiensis* strains are described as the causative agents of an emetic foodborne intoxication in the United Kingdom, involving pasta, mackerel, and mussels (Pheepakpraw et al., 2023[186]).

## Introductory Remarks – the Difficulty of Taxonomic Classification of the B. Cereus Group

*B. cereus sensu stricto *(*s. s.*) provides the name for the *B. cereus* group, also referred to as *B. cereus* *sensu lato *(*s. l.*). The members of this group exhibit significant genetic similarities, leading to repeated attempts to classify them as a single species (Ash et al., 1991[10]; Helgason et al., 2000[101]; Okinaka and Keim, 2016[180]; Stenfors Arnesen et al., 2008[214]). In contrast to this, there are significant differences, particularly in the formation of toxins, some of which are plasmid-encoded (Dietrich et al., 2021[56]; Okinaka and Keim, 2016[180]; Stenfors Arnesen et al., 2008[214]). Initially, species differentiation was based on phenotypic characteristics (Granum, 2007[88]), later on phylogenetic relationships determined by sequencing of individual genes or by multi-locus sequence typing (MLST) (Bavykin et al., 2004[12]; Bazinet, 2017[13]; Didelot et al., 2009[52]; Guinebretiére et al., 2008[93]; Okinaka and Keim, 2016[180]). Based on whole genome sequence typing, Kovac et al. (2016[128]) defined nine different phylogenetic clades and linked them to the production of enterotoxins for the first time. Beyond that, Carroll et al. (2020[37]) proposed a new taxonomic nomenclature for the *B. cereus* group, consisting of genomospecies, subspecies and biovar, taking into account genomic definitions, medical and industrial phenotypes, as well as historically relevant species names. The most prominent examples of this group are *Bacillus anthracis, *causative agent of anthrax (Pilo and Frey, 2018[188]), *B. thuringiensis*, which is used worldwide as a biopesticide (Biggel et al., 2022[22]; Chung et al., 2024[43]; Jouzani et al., 2017[120]), *Bacillus cytotoxicus* (Cairo et al., 2022[32]; Guinebretiére et al., 2013[91]; Lund et al., 2000[151]) and, obviously, *B. cereus*. For an accurate and successful risk assessment of the toxic potential, particularly in the context of foods, a classification based solely on taxonomy is unlikely to be effective. A current review article summarises the different methods applied to distinguish the members of the *B. cereus* group. Furthermore, advantages and disadvantages of five different taxonomic frameworks are discussed, which recognise different numbers of *B. cereus *group species, ranging from 5 to 40 (Carroll et al., 2022[35]).

## Distribution of Toxin Genes within the B. Cereus Group

Due to its ubiquitous nature, *B. cereus* has been detected in a broad range of foods in studies conducted across the world (Dietrich et al., 2021[56]). Often, species differentiation or taxonomic/phylogenetic classification were not performed, primarily, as the focus was set on determining the toxic potential by identifying toxin genes. In this context, it is also referred to as “presumptive *B. cereus*” (International Organization for Standardization, 2004[106]). Generally, 85-100 % of the tested isolates harbour the *nheABC* operon, 40-70 % the *hblCDA(B)* operon, 40-70 % *cytK-2*, and 0 % *cytK-1*. In addition, only few isolates carry the *ces* gene cluster (Dietrich et al., 2021[56]). Depending on their combination, isolates are allocated to toxin profiles A-G: A (*nhe*, *hbl*, *cytK*-*2*), B (*nhe*, *cytK-2*, *ces*), C (*nhe*, *hbl*), D (*nhe*, *cytK-2*), E (*nhe*, *ces*), F (*nhe*), G (*cytK-1*) (Ehling-Schulz et al., 2006[63]).

In contrast to plasmid-encoded anthrax, cereulide or insecticidal toxins, genes encoding the enterotoxins Nhe, Hbl and CytK are located on the bacterial chromosome, and thus, especially *nheABC* can be found in further *B. cereus* group members. *B. anthracis* for instance carries these enterotoxin genes, but their expression is silenced due to a point mutation in the *plcR* gene resulting in an unfunctional transcriptional regulator (Mignot et al., 2001[169]). *B. thuringiensis *has also been shown to not only carry the *nhe* and *hbl* operons as well as *cytK-2*, but also to express significant amounts of toxin under various cultivation conditions, which prompted a vigorous debate about the potential health risks associated with the widely used biopesticidal strains (Biggel et al., 2022[22]; Chattopadhyay and Banerjee, 2018[41]; EFSA, 2016[61]; Johler et al., 2018[118]; Jouzani et al., 2017[120]; Schwenk et al., 2020[205]). Enterotoxin genes were also detected in further species in- and outside of the *B. cereus* group, such as *Bacillus mycoides*, *Bacillus pseudomycoides*, *Bacillus toyonensis*, *Bacillus pasteurii*, *Bacillus amyloliquefaciens*, and *Bacillus circulans* (Dietrich et al., 2021[56]).

Occasionally, uncommon hybrid forms are detected, such as *B. cereus* strains responsible for anthrax-like illness (Marston et al., 2016[157]; Saikia et al., 2019[199]), or the above-mentioned emetic *B. thuringiensis* isolates (Pheepakpraw et al., 2023[186]). Böhm et al. (2015[23]) found proof for horizontal transfer of the *hbl*, *cytK* and *plcR *genes, frequent deletion of both toxins and duplication of *hbl*, and concluded this frequent exchange to be an important mechanism of virulence evolution within the *B. cereus* group. Furthermore, Carroll and Wiedmann (2020[36]) highlighted dynamic and ongoing acquisition and loss of cereulide synthetase genes resulting in a switch between diarrhoeal and emetic foodborne pathogens within group III *B. cereus* *s. l.* isolates. Finally, it should be noted that the simple presence of toxin genes does not allow reliable conclusions regarding the toxic potential or health risk of an isolate. Instead, it is determined by complex transcriptional and post-transcriptional regulation, which can vary greatly among strains.

## Enterotoxins and Diarrhoeal Syndrome

The diarrhoeal syndrome is caused by the three different pore-forming enterotoxins haemolysin BL (Hbl), non-haemolytic enterotoxin (Nhe) and cytotoxin K (CytK). Hbl and Nhe are tripartite toxins consisting of the protein components Hbl L2, L1 and B (Beecher and MacMillan, 1990[16], 1991[15]), and Nhe A, B and C (Lund and Granum, 1996[152]), respectively. CytK is a single protein (Lund et al., 2000[151]).

### Genetic organisation and gene regulation

**Figure 3:**
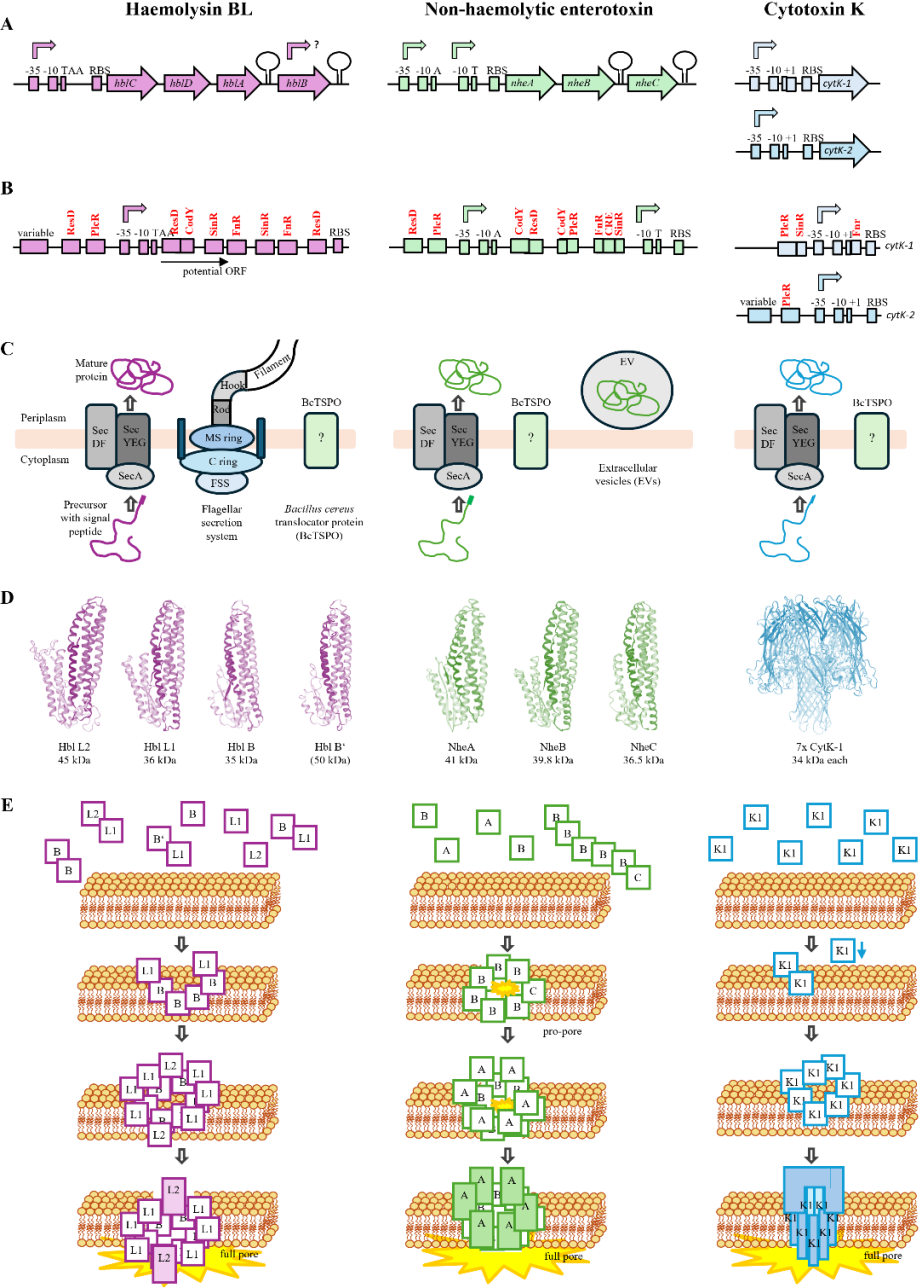
Properties of the *B. cereus *enterotoxins Hbl (haemolysin BL), Nhe (non-haemolytic enterotoxin) and CytK (cytotoxin K): (A) Structure of the *hbl* and *nhe* operons as well as the *cytK-1* and *cytK-2* genes including promoter regions (based on Dietrich et al., 2021). (B) Detailed depiction of the regions upstream of these operons/genes including transcriptional regulator binding sites (based on Böhm et al., 2016). The gene and promoter schematics were not drawn to scale. (C) Description of the secretory elements involved in enterotoxin secretion (Buchacher et al., 2023; Duport and Armengaud, 2025; Fagerlund et al., 2010; Ghelardi et al., 2007; Mazzantini et al., 2020; Salvetti et al., 2007; Tsukazaki, 2018; Vörös et al., 2014). (D) Proteinaceous enterotoxin components and their corresponding molecular weights. Protein structures were modelled using SWISS-MODEL (Waterhouse et al., 2018). Hbl B: accession 2NRJ, Hbl L1: accession 7NMQ, NheA: accession 4K1P, Hbl L2, NheB and NheC: modelled using the corresponding amino acid sequences from *B. cereus *strain F837/76 and 7NMQ as template due to highest coverage. Hbl B': Modelled using 2NRJ as template. The C-terminal part not consistent with Hbl B could not be modelled. Further, the homo-heptameric structure of the CytK-1 pore is shown, modelled using the corresponding amino acid sequence from *B. cereus *strains NVH 319-98 and 8RJ8 (Sauciuc et al., 2024) as template. Signal peptides for secretion were neglected. (E) Schematic depiction of the mode of action (pore formation) of the enterotoxins, adapted from Dietrich et al. (2021).

The genes *hblC, hblD *and *hblA, *which encode Hbl L2, L1 and B, respectively, are organised in an operon (Ryan et al., 1997[197]) (Figure 3A(Fig. 3); References in Figure 3: Buchacher et al., 2023[29]; Dietrich et al., 2021[56]; Duport and Armengaud, 2025[57]; Fagerlund et al., 2010[71]; Ghelardi et al., 2007[84]; Mazzantini et al., 2020[162]; Salvetti et al., 2007[200]; Sauciuc et al., 2024[202]; Tsukazaki, 2018[221]; Vörös et al., 2014[225]; Waterhouse et al., 2018[231]). The majority of *hbl*-positive isolates also bears *hblB*, encoding a fourth protein component, Hbl B' (Heinrichs et al., 1993[100]). Generally, sequence similarities point to the development of the whole *hbl* operon from duplication of a single precursor gene (Økstad et al., 1999[181]). Its transcription begins upstream of *hblC* and *hblCDA* are co-transcribed (Ryan et al., 1997[197]). Upstream of *hblB*, a stem loop as possible transcriptional terminator and a possible new promoter structure were identified (Clair et al., 2010[45]; Sastalla et al., 2013[201]). The *nheABC* operon encodes Nhe A, B and C (Granum et al., 1999[89]) (Figure 3A(Fig. 3)). All three genes are co-transcribed (Lindbäck et al., 2004[141]), but similarly to *hblB*, stem loops were identified in the intergenic region between *nheB* and *nheC* as well as downstream of *nheC *(Granum et al., 1999[89]). Upstream of *nheA*, two promoter regions containing two PlcR binding sites exist. These are part of uncommonly long 5' untranslated regions present upstream of the *hbl* and *nhe* operons but lacking upstream of *cytK* (Böhm et al., 2016[24]) (Figure 3B(Fig. 3)). The latter encodes the single protein toxin CytK (Figure 3A(Fig. 3)). Sequence analyses revealed two highly homologue variants, *cytK-1 *and *cytK-2 *(Fagerlund et al., 2004[73]; Guinebretiére et al., 2006[92]). Only few strains, mainly isolated from potato products or flour and just recently, from edible insects, produce the toxic CytK-1 variant, which are classified as a separate species, *B. cytotoxicus *(Cairo et al., 2022[32]; Contzen et al., 2014[48]; Etter et al., 2024[68]; Guinebretiére et al., 2013[91]; Heini et al., 2018[99]; Kone et al., 2019[125]; Mohammadpour et al., 2025[172]). Although *cytK-1* is highly conserved within this species, recent whole genome sequence analyses reveal considerable chromosomal and plasmidial intra-species diversity (Fayad et al., 2021[75]). The *cytK-2* gene is frequently found in *B. cereus* isolates; however, it seems not to be relevant to the diarrhoeal disease (Castiaux et al., 2015[38]; Jessberger et al., 2019[115]; Jessberger et al., 2015[116]).

The promoter regions of the *hbl* and *nhe* operons as well as *cytK-1* and *cytK-2* bear binding sites (known as PlcR-boxes with the conserved sequence TATGNANNNNTNCATA) for the pleiotropic transcriptional regulator PlcR (Figure 3B(Fig. 3)), which acts as an activator for its regulon at the transition from exponential to stationary growth phase via a well-investigated mechanism responding to cell density (Agaisse et al., 1999[2]; Gohar et al., 2002[86], 2008[85]; Lereclus et al., 1996[134], 2000[135]). Herein, the signalling peptide PapR plays a particularly important role as part of the quorum-sensing system (Bouillaut et al., 2008[25]; Slamti and Lereclus, 2002[211]). This peptide is exported, and with the help of secreted neutral protease B (NprB) and oligopeptide permease OppABCDF subsequently processed and re-imported as heptapeptide PapR_7_. PlcR-PapR complexes can then bind to the PlcR-boxes and activate gene expression (Bouillaut et al., 2008[25]; Gominet et al., 2001[87]; Pomerantsev et al., 2009[190]; Slamti and Lereclus, 2002[211]). *hbl*, *nhe* and *cytK-1* promoter regions furthermore show binding sites for Fnr and SinR (Böhm et al., 2016[24]) (Figure 3B(Fig. 3)). Fnr (fumarate and nitrate reductase) is a redox regulator activating enterotoxin gene expression in response to carbohydrates and oxidoreduction potential (Messaoudi et al., 2010[167]; Zigha et al., 2007[241]). SinR, which - together with its antagonists - is primarily involved in biofilm formation and motility, was proven to influence *hbl* transcription (Fagerlund et al., 2014[70]; Milton et al., 2020[171]; Newman et al., 2013[178]). Additionally, the *hbl* and *nhe *upstream regions harbour different ResD binding sites (Figure 3B(Fig. 3)), which is the response regulator interacting with sensor kinase ResE in response to oxidoreduction potential (Duport et al., 2006[60]). ResD and Fnr have mutual influence on each other (Esbelin et al., 2009[67]). Palindromic catabolite responsive elements (CRE) have also been identified upstream of *hbl *and *nhe* (Böhm et al., 2016[24]; Stenfors Arnesen et al., 2008[214]; van der Voort et al., 2008[222]) (Figure 3B(Fig. 3)). These are binding sites to CcpA, a transcriptional regulator repressing *hbl* and *nhe* expression under different conditions including glucose surplus (Duport et al., 2004[59]; Ouhib-Jacobs et al., 2009[183]; Ouhib et al., 2006[182]; van der Voort et al., 2008[222]). Finally, one (*hbl*) and two (*nhe*) CodY binding site were also found (Böhm et al., 2016[24]) (Figure 3B(Fig. 3)). Under nutrient surplus, the regulon of this pleiotropic transcriptional regulator is repressed. As nutrients become limited, derepression occurs. Affected genes are involved in catabolism, chemotaxis and motility, as well as virulence (Brinsmade, 2017[28]; Lindbäck et al., 2012[143]). CodY affects the PlcR-PapR quorum sensing system as well as the *hbl* and *nhe* operons by direct binding (Böhm et al., 2016[24]; Slamti et al., 2015[210] ). The regulator is phosphorylated at serin 215 by the Hanks kinases PrkC and YbdM, which impedes DNA binding (Kortebi et al., 2025[127]). Böhm et al. (2016[24]) found that, in contrast to PlcR, the CodY binding sites show a much higher heterogeneity within the *B. cereus* group. This might lead to varying binding intensities of this nutrient-responsive roadblock repressor, and thus, to strain-specific intensities of enterotoxin production (Böhm et al., 2016[24]). Furthermore, Prince and Kovac emphasise in their minireview, that although the upstream regions of the *hbl* and *nhe* operons show binding sites for the same transcriptional regulators, they are differentially regulated. This might be due to different modes of action of regulators, once as activators for *nhe*, once as repressors for *hbl*, stronger binding of repressors upstream of *hbl*, stronger binding of activators upstream of *nhe*, or further unidentified transcriptional regulators (Prince and Kovac, 2022[192]). Further studies suggest an involvement of the OhrRA (organic hydroperoxide resistance) system (Clair et al., 2013[44] and the transcriptional regulator RpoN (sigma 54) (Hayrapetyan et al., 2015[97]) on *hbl* and *nhe* expression, as well as a new, yet unknown regulatory mechanism for *hblB* (Clair et al., 2010[45]; Jessberger et al., 2019[112]). In summary, it has been shown that enterotoxin gene expression depends on cellular growth phase and energy status, temperature, oxygen tension, nutrient availability, and host factors (Dietrich et al., 2021[56]; Jessberger et al., 2019[112]; Jessberger et al., 2017[117]; Prince and Kovac, 2022[192]; Vyklicka et al., 2025[226]). Further, Meng et al. (2025[166]) found that enterotoxin gene expression can be decreased by environmental stress factors such as temperature, pH and salt concentrations.

### Enterotoxin secretion and translocation

On the one hand, it is believed that the Hbl and Nhe enterotoxin components as well as CytK are released from the cell via the Sec translocation pathway, as all of them contain N-terminal signal peptides for secretion. SecA (ATPase), SecDF (translocation factor) and SecYEG (channel) assemble this pathway (Beckwith, 2013[14]; Tsukazaki, 2018[221]) (Figure 3C(Fig. 3)). Experimentally, a loss of Hbl B secretion was shown after signal peptide modification, as well as reduced enterotoxin secretion after addition of SecA inhibitor sodium azide (Fagerlund et al., 2010[71]). A *secDF* knock out mutant showed lower amounts of Hbl, Nhe, CytK and phospholipase C in the secretome as well as reduced virulence and motility compared to the wild type (Vörös et al., 2014[225]). On the other hand, especially Hbl secretion has been linked to the flagellar secretion system (Figure 3C(Fig. 3)). A connection was established between swarming motility and Hbl secretion (Ghelardi et al., 2007[84]), and an *flhA* (integral transmembrane protein) deletion compromised Hbl secretion as well as *hblC* transcription (Bouillaut et al., 2005[26]; Ghelardi et al., 2002[83], 2007[84]). Impaired Hbl secretion was also observed after deletion of *flhF* (signal recognition particle-like GTPase). Interestingly, enhanced levels of Nhe and further virulence factors were detected (Mazzantini et al., 2016[161]; Salvetti et al., 2007[200]). It has further been shown that FlhF homodimers can bind Hbl L2 with the purpose of directing it to the plasma membrane for secretion (Mazzantini et al., 2020[162]). In contrast to that, it was recently proven that a *fliF* (flagellar MS-ring) deletion compromises motility and adhesion, but not Hbl secretion (Mazzantini et al., 2024[163]).

Apart from these two secretion systems, Duport et al. (2020[58]) compared the dynamics of the proteome and exoproteome in *B. cereus* AH187, an emetic strain which also produces Nhe, at 16 and 30 °C growth temperature. The authors concluded that exotoxins such as Nhe or cereolysin O are a major part of temperature-induced proteome changes and are important in controlling homeostasis of the cellular proteome and exoproteome during cold stress (Duport et al., 2020[58]). In a similar approach, comparing *B. cereus *ATCC 14597 and a Δ*tspo *mutant, it was found that this translocator protein (BcTSPO, Figure 3C(Fig. 3)) significantly influences cellular metabolism and stress response by re-structuring the cellular proteome and exoproteome. It was particularly noticeable that the enterotoxins Hbl, Nhe and CytK were significantly reduced in the exoproteome in the absence of BcTSPO, while the level of the above-mentioned cereolysin O increased (Duport and Armengaud, 2025[57]). It has also recently been discovered that extracellular vesicles can play an important role in virulence of *B. cereus*. It has been shown for strain NVH0075-95 that these sphere-shaped, membranous entities, which are released into the extracellular space by a yet not fully understood mechanism (Briaud and Carroll, 2020[27]), contain virulence factors such as sphingomyelinase, phospholipase C, and the three Nhe components (Buchacher et al., 2023[29]) (Figure 3C(Fig. 3)). Overall, *B. cereus* not only exhibits highly variable enterotoxin gene transcription, but also various, potentially interrelated secretion mechanisms, which may further account for the high toxin- and strain-specific differences.

### Mode of action

The first studies conducted on Hbl activity described dermonecrotic, haemolytic and cytotoxic properties, activity in vascular permeability tests, as well as fluid accumulation in rabbit ileal loops (Beecher and Macmillan, 1991[15]; Beecher et al., 1995[18], 2000[17]; Beecher and Wong, 1994[19]; Lund and Granum, 1997[153]). The three Hbl components have a size of 45 kDa (L2), 36 kDa (L1) and 35 kDa (B) (Beecher and Macmillan, 1991[15]) (Figure 3D(Fig. 3)), but variable homologues can exist (Beecher and Wong, 2000[20]; Schoeni and Wong, 1999[203]). All three components are required for biological activity (pore-formation) of the toxin complex, as well as a specific binding order and a defined concentration ratio. Even before reaching the target cells, Hbl components can form complexes in solution, mainly B-B, B-L1, L2-L1, and only weakly, B-L2 (Jessberger et al., 2020[113]; Tausch et al., 2017[216]). This preparatory configuration facilitates rapid pore formation (Jessberger et al., 2014[109]; Jessberger et al., 2019[114]). The required binding order is B-L1-L2 (Jessberger et al., 2019[114]; Sastalla et al., 2013[201]), whereby the binding of Hbl B to the target cell surface is enhanced by its complexation with Hbl L1 (Jessberger et al., 2019[114]). In addition, the concentration ratio of the components plays a decisive role. Ratios of L2:L1:B = 1:1:10 and 10:1:10 lead to most rapid pore formation, thus, excess of Hbl B accelerates, excess of Hbl L1 decelerates pore formation, while the Hbl L2 levels seem not to be crucial (Jessberger et al., 2019[114]) (Figure 3E(Fig. 3)). Another study, using a Chinese hamster ovary cell-free system for the synthesis of the Hbl components, mainly confirmed these results, but further claimed pore-forming activity of a “Hbl L1-L2 pre-pore-complex” on Caco-2 cells (Ramm et al., 2021[195]). Contrary to that, a further study involving the production of His-tagged recombinant Hbl components in a prokaryotic expression system, did not determine haemolytic or cytotoxic activity of any two components (Chen et al., 2023[42]).

The Hbl pores in the plasma membrane are rather small with a probable channel diameter of approx. 1.2 nm (Beecher and Wong, 1997[21]; Jessberger et al., 2020[113]). Further, these moderately cation selective pores are rather unstable and of limited lifespan (Jessberger et al., 2020[113]). The putative fourth component of the Hbl complex, Hbl B', has first been detected in the secretome of *B. cereus* ATCC 14579 (Clair et al., 2010[45]), and later, using Western blot analyses, in concentrations from 0.5 to 4.8 ng/µl in 10 further enteropathogenic strains (Jessberger et al., 2022[111]). With its approx. 50 kDa, the protein is larger than Hbl B. Despite sequence similarities, it is not able to perform or enhance the function of Hbl B. On the contrary, when used in excess, Hbl B' reduces cytotoxic and haemolytic activity of Hbl (Jessberger et al., 2022[111]). Further, the protein shows no significant influence on the binding of Hbl B to the target cell surface; Hbl B' rather interacts directly with L1, thereby inhibiting its complex formation with B. Hbl B' can therefore be attributed an important regulatory function in Hbl complex formation, which is the balanced orchestration of Hbl B-L1 complexes and the corresponding free subunits (Jessberger et al., 2022[111]).

The three Nhe components have a size of 41 kDa (A), 39.8 kDa (B) and 36.5 kDa (C) (Granum et al., 1999[89]) (Figure 3D(Fig. 3)), but other variants may also occur (Stenfors Arnesen et al., 2008[214]). Due to structural similarities, both Hbl and Nhe were allocated to the Cytolysin A (ClyA) superfamily of α-helical pore forming toxins, which was ultimately confirmed by their crystal structure (Fagerlund et al., 2008[72]; Ganash et al., 2013[82]; Madegowda et al., 2008[154]; Phung et al., 2012[187]; Worthy et al., 2021[234]). Despite all similarities, the components of the two enterotoxin complexes cannot be interchangeably used or complement the respective other pore (Sastalla et al., 2013[201]). In contrast to Hbl, Nhe is known to form highly stable complexes of approx. 620 kDa between B and C, which can form pre-pores, transmembrane channels of approx. 2 nm diameter, in a suitable concentration ratio (NheB:C = 10:1) (Heilkenbrinker et al., 2013[98]; Zhu et al., 2016[240]). Nevertheless, all three Nhe components are necessary for maximum biological activity, with an optimum concentration ratio of NheA:B:C = 10:10:1 and a specific binding order (Lindbäck et al., 2004[141], 2010[142]) (Figure 3E(Fig. 3)). For completion of the whole pore, NheA, which cannot interact with the other components in solution, binds to cell-bound NheB and undergoes massive conformational changes (Didier et al., 2012[53], 2016[54]). The complete pore is large, > 2.8 nm, and stable, similarly to ClyA, and has a conductance of approx. 18 nS on planar lipid bilayers (Fagerlund et al., 2008[72]; Ramm et al., 2020[194] ). Similarly to Hbl and ClyA, it is cation-selective (Fagerlund et al., 2008[72]; Jessberger et al., 2020[113]; Ludwig et al., 1999[150]).

In contrast to Hbl and Nhe, CytK is a single protein toxin of approx. 34 kDa (Figure 3D(Fig. 3)). It is assigned to the family of β-barrel pore-forming toxins, which are typically present as monomers in solution, attach to the target cell membrane, oligomerise into heptamers, undergo conformational changes, and ultimately form the mushroom-shaped pore (Andreeva et al., 2006[9], 2007[8]; Cairo et al., 2022[32]; Menestrina et al., 2001[165]) (Figure 3E(Fig. 3)). CytK proved to be pore-forming, haemolytic, dermonecrotic and cytotoxic, with CytK-1 showing an approx. 80 % enhanced activity compared to CytK-2 (Fagerlund et al., 2004[73]; Hardy et al., 2001[95]). The pores are weakly anion selective with a predicted minimum diameter of approx. seven Å (Hardy et al., 2001[95]). Recently, Sauciuc et al. (2024[202]) showed that during electroosmotically driven translocation of polypeptides, blob-like structures are shaped inside nanopores, including those of CytK. These blobs seem to interfere with polypeptide transportation and inhibit the targeting of certain amino acids (Sauciuc et al., 2024[202]). However, it has recently been shown that CytK-1 appears to be less toxic than previously thought. Various *B. cytotoxicus* isolates, mainly obtained from potato products, showed little to no cytotoxicity towards Vero cells (Burtscher et al., 2021[30]; Etter et al., 2024[68]). Nevertheless, cytotoxicity tests on Caco-2 cells using *cytK-1* knockout mutants proved that CytK-1 remains the major pathogenicity factor in *B. cytotoxicus *(Kone et al., 2021[126]).

### Response of target cells

The three main *B. cereus* enterotoxins act on a wide range of target cell lines and tissue with variable strength (Dietrich et al., 2021[56]). This and the non-interchangeability of the Hbl and Nhe protein components (see above), suggest different receptor types on the target cell surface. For Hbl, lipopolysaccharide (LPS)-induced tumour necrosis factor (TNF)-α factor (LITAF) was identified as the main receptor, using a genome-wide CRISPR-Cas9 knockout screen in RAW276.4 mouse macrophages. Additionally, the LITAF-like protein cell death-inducing P53 target 1 (CDIP1) was determined as a second, alternative receptor in LITAF-deficient cells. Cell lines as well as mice lacking LITAF and/or CDIP1 showed resistance towards Hbl-mediated toxicity (Liu et al., 2020[144]). Since LITAF functions as an activator of inflammation and is present in various human cell types, tissues, and organs, it is speculated that targeting this receptor via Hbl is part of the strategy of *B. cereus* to cause cell death responses in the host (Enosi Tuipulotu et al., 2021[66]). The receptor for Nhe, which likely differs from LITAF and CDIP1, has not yet been identified. Instead, it has been shown that the above-mentioned Nhe-containing extracellular vesicles use cholesterol-rich domains and dynamin-mediated endocytosis to connect with intestinal epithelial cells, resulting in internalisation of Nhe and prolonged cytotoxicity. These vesicles also seem to provoke an inflammatory response in human monocytes (Buchacher et al., 2023[29]). A specific receptor has not yet been identified for CytK either. However, it is understood that β-barrel pore-forming toxins typically bind to the target cell membrane through interaction with liposomes (Andreeva et al., 2007[8]; Menestrina et al., 2001[165]; Song et al., 1996[212]).

Shortly summarised, the *B. cereus* enterotoxins form pores leading to damage and osmotic lysis of intestinal epithelial cells, injury of microvilli, and consequently diarrhoea (Enosi Tuipulotu et al., 2021[66]). The cellular biological processes underlying this are vastly more complex and have not yet been fully elucidated. First, it has been demonstrated that Nhe pore formation causes calcium influx, oxidative stress and activation of apoptosis signal-regulating kinase 1 (ASK1) and p38 MAPK in Vero cells. Subsequent activation of caspase-8 and caspase-3 triggers apoptosis (Liu et al., 2017[145]). Newer studies showed that potassium efflux, caused by both Hbl and Nhe pores, leads to inflammasome-mediated mortality of macrophages (Fox et al., 2020[76]; Mathur et al., 2019[160]). As part of the innate immune system, this protein complex consists of a sensor protein, an apoptosis-associated-speck-like adaptor protein with a caspase recruitment and activation domain, and the cysteine protease caspase-1 (Enosi Tuipulotu et al., 2021[66]). In this case, the sensor protein is NLRP3 (NOD-, LRR- and pyrin domain-containing protein 3). Activation of the inflammasome leads to secretion of the proinflammatory cytokines interleukin-1β and interleukin-18, as well as cleavage of the pore-forming protein gasdermin D, causing pyroptosis, an inflammatory form of cell death (Enosi Tuipulotu et al., 2021[66]; Fox et al., 2020[76]; Mathur et al., 2019[160]). Not surprisingly, Hbl-induced NLRP3 inflammasome activation occurs quicker compared to Nhe (Fox et al., 2020[76]; Mathur et al., 2019[160]). This corresponds with the above-mentioned pore sizes as well as with the higher velocity of Hbl pore formation (Jessberger et al., 2014[109]). It is further speculated that different receptor densities on the target cell surface may also play a role (Enosi Tuipulotu et al., 2021[66]). Based on these studies, Zhao et al. also demonstrated induction of NLRP3/gasdermin D-dependent pyroptosis by pathogenic *B. cereus* H2 from a deep-sea cold seep as well as by purified, recombinant CytK (Zhao et al., 2021[237]; Zhao and Sun, 2022[238]).

As a further consequence of *B. cereus* infections, Jovanovic and Rajkovic (2023[122]) showed bioenergetic changes in Caco-2 cells, described as a more energetic phenotype caused by increased flux through oxidative phosphorylation and glycolysis, as well as higher ATP levels. As our own latest intestinal model, we exposed a 9:1 Caco-2/HT29-MTX co-culture to *B. cereus* culture supernatants as well as purified enterotoxin leading to enterotoxin-, dose-, differentiation- and time-dependent decline in trans-epithelial electrical resistance (TEER), as well as increased short-circuit currents (Isc) consistent with toxin-induced alterations in epithelial ion transport. Furthermore, the co-culture demonstrated partial recovery after toxin removal. Total transcriptome analyses revealed a specific time- and dose-dependent host response programme. Lethal toxin doses reduced proliferation- and cell cycle-related gene expression, while TNF/NF-κB-driven inflammation, MAPK-associated stress signalling, and cell-fate programmes involving the p53-mediated DNA-damage/stress response as well as regulated cell death (apoptosis) were up-regulated (Albahri et al., 2026[4]). Using a similar methodology but pursuing different goals, Fang et al. (2025[74]) found 937 differentially expressed genes in splenic cells of Chinese soft-shelled turtles after *B. cereus* infection and identified relevant pathways involved in the response to this infection.

## Cereulide and Emetic Syndrome

**Figure 4:**
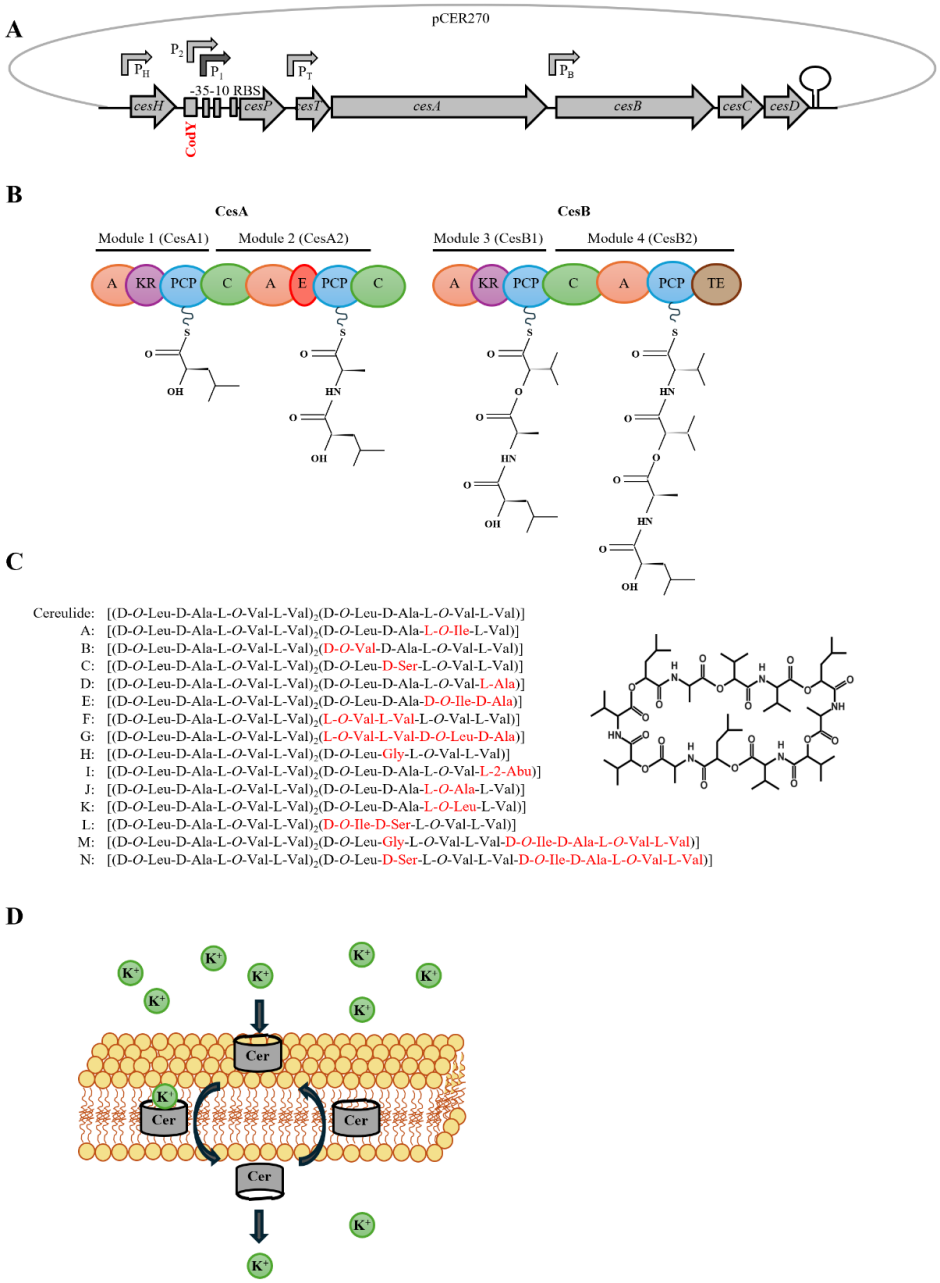
Properties of the emetic toxin cereulide of *B. cereus*: (A) Structure of the *ces* operon including promoters and the CodY binding site located on megaplasmid pCER270 (based on Dietrich et al., 2021; Frenzel et al., 2012). *cesH*: hydrolase/esterase, *cesP*: phosphopantetheinyl transferase, *cesT*: type II thioesterase, *cesA* and *cesB*: structural cereulide synthetase genes, *cesC* and *cesD*: ABC transporter. Polycistronic transcription of the *ces* operon is driven by main promoter P1. (B) Schematic representation of cereulide synthesis by non-ribosomal peptide synthetase (NRPS) (based on Alonzo et al., 2015; Jovanovic et al., 2021). A: adenylation domain, KR: ketoreductase domain, PCP: peptidyl carrier protein, C: condensation domain, E: epimerisation domain, TE: thioesterase domain. (C) Ring-shaped structure of the dodecadepsipeptide cereulide and amino acid sequences of cereulide and isocereulides (based on Marxen et al., 2015a; Walser et al., 2021, 2022). (D) Mode of action of cereulide (based on Jovanovic et al., 2021). Cereulide binds and moves K^+^ ions through the lipid bilayer according to the electrochemical potential.

The emetic syndrome was first described in 1976 after cases of food poisoning occurred in the United Kingdom following the consumption of cooked rice (Melling et al., 1976[164]). It is caused by cereulide, a highly stable cyclic dodecadepsipeptide with a molecular weight of 1.2 kDa and the repeated amino acid sequence [D-*O*-Leu-D-Ala-L-*O*-Val-L-Val]_3_ (Agata et al., 1994[3]) (compare Figure 4(Fig. 4); References in Figure 4: Alonzo et al., 2015[5]; Dietrich et al., 2021[56]; Frenzel et al., 2012[79]; Jovanovic et al., 2021[121]; Marxen et al., 2015[158]; Walser et al., 2021[228]; Walser et al., 2022[227]).

### Genetic organisation and gene regulation of cereulide synthesis

The toxin is synthesised by a non-ribosomal peptide synthetase (NRPS). All genes involved in this process are encoded in what is known as the *ces* gene cluster, which is located on a pX01-like megaplasmid called pCER270 (Ehling-Schulz et al., 2005[64], 2006[62]). The 24 kbp gene cluster encodes seven enzymes, including a hydrolase/esterase (CesH), a phosphopantetheinyl transferase (CesP), a type II thioesterase (CesT), the structural NRPS core (CesA and CesB, assembling amino acids into the cyclic peptide) as well as an ABC transporter consisting of CesC and CesD (Ehling-Schulz et al., 2005[64], 2006[62]; Gacek-Matthews et al., 2020[81]). From the main promoter P1, six of the seven genes, *cesPTABCD*, are transcribed as a single 23-kb polycystron, mostly at late exponential growth phase. The role of additional promoters is not yet understood (Frenzel et al., 2012[79]; Lücking et al., 2009[148]) (Figure 4A(Fig. 4)). *cesH*, whose corresponding protein inhibits cereulide synthesis by a rather indirect mechanism, is expressed from its own promoter structure (Lücking et al., 2015[149]; Tian et al., 2019[219]). The *ces* gene cluster is not controlled by PlcR, but indeed by CodY (see above) and the transition state transcriptional regulator AbrB, as well as Spo0A (Frenzel et al., 2012[79]; Lücking et al., 2009[148]). Additionally, the plasmid-encoded, ArsR/SmtB family transcriptional regulator PagRBc was identified, which represses cereulide synthetase gene expression (Kalbhenn et al., 2022[123]). Moreover, posttranscriptional regulation plays an important role in cereulide synthesis, for example activation of NRPS by plasmid (CesP) and chromosomal 4´-phosphopanthetheinyl transferases (Ehling-Schulz et al., 2006[62]; Lücking et al., 2015[149]; Walsh, 2016[229]), or the essential part of ABC transporter CesCD in cereulide biosynthesis (Gacek-Matthews et al., 2020[81]). A role in cereulide production was also demonstrated for a flagellar hook protein after the corresponding gene *flgE* was knocked out (Li et al., 2022[139]). Transferring pCER270 from emetic to phylogenetically distant *B. cereus* group strains demonstrated transcriptional cross-regulation between the megaplasmid and the host genome, for instance regarding carbohydrate metabolism and sporulation genes expression, as well as pCER270 encoded genes (Nevers et al., 2023[176]).

Several studies have further shown that extrinsic parameters, such as temperature, oxygen content, a_W_, pH, food matrix or food additives influence levels and even isoforms of produced cereulide (Frenzel et al., 2011[80]; Häggblom et al., 2002[94]; Kranzler et al., 2016[131]; Messelhäusser et al., 2014[168]; Rouzeau-Szynalski et al., 2020[196]; Wang et al., 2024[230]; Yang et al., 2023[235]). It is known that branched-chain amino acids enhance binding of CodY to the *ces* promoter region (Frenzel et al., 2012[79]) and just recently, Li et al. (2026[137]) demonstrated that isoleucine regulates cereulide biosynthesis via folate metabolism. Some phytochemicals, such as gingerol and curcumin, were shown to inhibit not only growth, but also cereulide production of emetic *B. cereus* at concentrations ≤ 0.01 % (Kranzler et al., 2021[130]). It has further been suggested that lactose can function as a signal molecule to regulate cereulide production by repressing *ces* gene expression, possibly due to inhibition of the Spo0A regulator (Zheng et al., 2024[239]). Modelling growth and cereulide production at different temperatures for microbiological food safety assessment, Ellouze et al. (2021[65]) concluded that cereal-based food matrices highly support cereulide production, followed by culture medium and dairy-based matrices with intermediate influence, and meat- and vegetable-based matrices with lowest cereulide formation. Similarly, Buss da Silva et al. (2022[31]) modelled time to first cereulide quantification, maximum growth rates and cereulide production rates as a function of temperature in an intermediate dairy wet-mix. They admitted, however, that the models cannot be easily transferred to unrelated food matrices, particularly with regard to cereulide production (Buss da Silva et al., 2022[31]). Moreover, cereulide production in an insect cadaver has been shown, allowing the conclusion that the toxin might be of ecological relevance for a saprophytic lifestyle (Jenull et al., 2023[108]), but also raising the question if edible insects might be a source of cereulide intoxication. Apart from different food matrices and temperatures, it is known that cereulide production ability differs widely among *B. cereus* isolates (Carlin et al., 2006[34]; Frentzel et al., 2024[78]), and three classes of cereulide producers have been defined: low (< 1-50 ng/g bacterial mass), medium (> 50-500 ng/g), and high (> 500-1600 ng/g) (Stark et al., 2013[213]). So far, this could be explained neither by sequence alterations in *cesHPTABCD* or the genes encoding the corresponding regulatory proteins, nor population structure and phylogeny of isolates subjected to whole genome sequencing. Nevertheless, the involvement of further associated proteins was suggested, which still needs to be verified (Frentzel et al., 2024[78]).

### Non-ribosomal cereulide synthesis

Of the seven Ces enzymes (see above), CesA and CesB comprise the actual cereulide synthetase, while CesA is responsible for D-Ala-D-*O*-Leu synthesis, and CesB for L-Val-L-*O*-Val (Marxen et al., 2015[159]; Rouzeau-Szynalski et al., 2020[196]) (see also Figure 4B(Fig. 4)). First, 4-methyl-2-oxopentanoic acid is converted into d-2-hydroxy carboxylic acid by the ketoreductase domain of CesA. Second, the acid is activated as adenosine monophosphate ester by the adenylation domain. The epimerisation domain epimerises L-Ala and another adenylation domain activates it. Subsequently, both monomers are attached to a peptidyl carrier protein and the free amine of D-Ala attacks on the D-*O*-Leu thioester. The condensation domain catalyses dimer formation (Jovanovic et al., 2021[121]). Similarly, L-Val-L-*O*-Val is synthesised by CesB. Then, another condensation domain facilitates the attack of D-*O*-Leu on the thioester-activated L-Val-L-*O*-Val, which transfers a tetradepsipeptide onto the PCP domain of CesA. In a last step, the thioesterase domain of CesB takes up the tetradepsipeptide and further chain elongation and macrocyclisation take place (Alonzo et al., 2015[5]; Jovanovic et al., 2021[121]; Magarvey et al., 2006[155]). Further, 4′-phosphopantetheinyl-transferase CesP activates NRPS and thioesterase CesT most likely removes misprimed monomers from the complex as part of its proofreading function. As mentioned above, CesCD is an ABC transporter most likely responsible for cereulide export, but also for self-resistance towards the toxin. CesH is probably involved in the right timing of cereulide synthesis (Lücking et al., 2015[149]; Rouzeau-Szynalski et al., 2020[196]).

Next to the initially discovered cereulide, NRPS also synthesises different isoforms, known as isocereulides, of which various have been identified to date (Figure 4C(Fig. 4)). An emetic *B. cereus* isolate is able to produce all cereulide isoforms. Nevertheless, strain- as well as temperature-dependent differences in their ratios were detected (Kranzler et al., 2016[131] ; Marxen et al., 2015[158][159]; Rouzeau-Szynalski et al., 2020[196]). A recent study showed stable ratios of cereulide and isocereulides A-N of approx. 1:9 both under standard laboratory growth conditions and in contaminated fried rice balls. In contrast to that, the ratios between the isocereulides varied time-dependently, indicating different kinetics of isocereulide production (Kranzler et al., 2024[132]). The structure of the isocereulides A-G has been resolved first (Marxen et al., 2015[158]; Pitchayawasin et al., 2004[189]; Stark et al., 2013[213]; Walser et al., 2021[228]). In comparison to cereulide, [D-*O*-Leu-D-Ala-L-*O*-Val-L-Val]_3_, the structure of highly toxic isocereulide A, for example, is [(D-*O*-Leu-D-Ala-L-*O*-Val-L-Val)_2_(D-*O*-Leu-D-Ala-L-*O*-Ile-L-Val)] (Walser et al., 2021[228]). Since then, the structures of the newly discovered isocereulides H-N have also been elucidated, all of which exhibit similar structural modifications compared to cereulide (Walser et al., 2022[227]) (Figure 4C(Fig. 4)).

### Mode of action and within host translocation

Cereulide was shown to restrain RNA and ATP synthesis, proliferation, as well as motility in eukaryotic cells. It was also suggested to interfere with the host's immune system (Agata et al., 1994[3]; Paananen et al., 2002[184]). Further, it is a potassium binding dodecadepsipeptide structurally resembling the ionophore valinomycin (Mikkola et al., 1999[170]; Suwan et al., 1995[215]; Teplova et al., 2006[217]) (compare Figure 4C(Fig. 4)). A classical ion-carrier transportation mechanism has been described: Cereulide-mediated transport across the lipid bilayer led to depolarisation and disturbance of the membrane potential in rat liver mitochondria by influx of K^+^ ions, which impairs ATP driving force and cellular respiration (Jääskeläinen et al., 2003[107]; Jovanovic et al., 2021[121]; Mikkola et al., 1999[170]; Teplova et al., 2006[217]) (Figure 4D(Fig. 4)). After slow release of K^+^ ions into the mitochondria, cereulide can diffuse back to the cytosol for continuous K^+^ uptake (Teplova et al., 2006[217]; Yang et al., 2023[235]). The different cereulide isoforms (see above) can vary significantly in their cytotoxic potential, reaching from 50 % to up to 8-fold compared to cereulide (Marxen et al., 2015[158]; Walser et al., 2022[227]). These variations can be explained by different membrane activity of the isocereulides (Marxen et al., 2015[158]). Constituting only 10 % of total toxins, isocereulides A-N were responsible for approx. 40 % of total cytotoxicity towards Hep2 cells in a recent study. Moreover, individual isocereulides showed higher or lower toxicity than cereulide, as well as additive and synergistic effects with the original toxin. This suggests that total toxicity originates from a mixture of cereulide and its variants, and that these variants must therefore also be included in routine food and clinical diagnostics (Kranzler et al., 2024[132]; Walser et al., 2022[227]). Until then, the effective dose leading to the onset of the emetic disease in humans is difficult to estimate, as it depends not only on cereulide, but also on its isoforms, as well as the susceptibility of the host, and the quantity of ingested food (Jovanovic et al., 2021[121]).

To better understand cereulide uptake, resorption and translocation within the host, the toxin was orally administered to piglets (Bauer et al., 2018[11]). While one part was rapidly excreted, another part was absorbed and accumulated in kidney, liver, muscles, fat and even brain tissues, leading to symptoms similar to critical human intoxications such as lethargy, seizures, and convulsions. It was concluded that the central nervous system is an important target for cereulide (Bauer et al., 2018[11]).

### Effects of cereulide intoxication

Since *B. cereus* is a ubiquitous soil inhabitant, it is reasonable to assume that cereulide plays an important ecological role. In a recent study, antibacterial activity of cereulide was tested and proven to be species-specific as well as enhanced with rising environmental K^+^ levels. Antifungal activity was K^+^-independent and only observed for *Candida albicans* and *Rhodotorula* species, while cereulide seemed to have no effect on *Nitrososphaera viennensis* belonging to the group of ammonia-oxidising archaea. Mimicking intoxication, cytotoxic activity towards Hep-2 cells increased with rising external K^+^ concentrations, while only cereulide and not external K^+^ concentrations showed an effect on the development of *Oesophagostomum dentatum* (nematode) larvae (Jenull et al., 2023[108]). Via experiments in a *Suncus murinus* animal model, cereulide has been found to interact with serotonin dependent receptors (5-HT3) stimulating the afferent vagus nerve and activating the vomiting centre in the *medulla oblongata* (Agata et al., 1994[3]). In relation to this, the cereulide dose necessary to cause emesis in *S. murinus* and rhesus monkeys has been determined at approx. 10 μg/kg body weight (Shinagawa et al., 1995[208]). The corresponding dose necessary to cause emesis in humans is not yet known.

Several studies demonstrated effects on gastrointestinal cells such as Caco-2 and HT-29. Among others, these were reduced non-mitochondrial respiration, maximal respiration, high-density lipoprotein (HDL) and ATP production, as well as inhibition of cell proliferation and the disturbance of intestinal barrier function after chronical, low dose cereulide exposure (Decleer et al., 2018[51]; Lin et al., 2021[140]; Rajkovic et al., 2014[193]; Yang et al., 2023[235]). Intestinal inflammation as well as apoptosis have also been observed in a mouse model mimicking chronic low-dose cereulide exposure. These resulted from endoplasmic reticulum (ER) stress IRE1/XBP1/CHOP pathway activation (Lin et al., 2021[140]). Dose-dependently, HepG2 (liver) cells showed reduction in maximum respiration and ATP production (Decleer et al., 2018[51]). Moreover, upon intraperitoneal cereulide exposure, mice showed pathological changes in the liver, such as hepatocyte degeneration, inflammation and necrosis, culminating in death of the mice at high doses (Yokoyama et al., 1999[236]). Modelling long-time cereulide exposure, Li et al. (2021[136]) found induction of oxidative stress and inflammation in mice, promoting apoptosis of liver and kidney cells. In detail, endoplasmic reticulum (ER) stress was activated via the pathways of inositol-requiring enzyme 1α (IRE1α)/Xbox binding protein (XBP1) and PRKR-like ER kinase (PERK)/eukaryotic translation initiation factor 2α (eIF2α). The authors further showed that this was due to accumulation of reactive oxygen species (Li et al., 2021[136]). In mouse pancreatic islets as well as in foetal porcine Langerhans islets, results ranged from reduced insulin production to death of MIN6 cells, depending on cereulide concentrations added (Hoornstra et al., 2013[103]; Vangoitsenhoven et al., 2014[223]; Virtanen et al., 2008[224]). Additionally, interference of cereulide with serotonin synthesis has been observed, leading to depression in animals. For this, cereulide either acts in the intestine on the synthesis of its precursor or, after crossing the blood-brain barrier, directly in the central nervous system (CNS) by inducing apoptosis (Bauer et al., 2018[11]; Lin et al., 2021[140]). Just recently, a complex neuronal mechanism of cereulide-induced retching as defence mechanism was discovered, involving Calbindin1-positive (Calb1^+^) neurons of the nucleus of the solitary tract (NTS). It was shown that cereulide directly modulates vagal sensory neurons which innervate Calb1^+^ NTS neurons. Further, it was demonstrated that two pathways, Calb1^NTS-PBNel^ and Calb1^NTS-Amb/RVLM^, mediate retching movement and nausea, respectively (Huo et al., 2024[104]). The toxin can also have a significant impact on the immune system, which was shown by loss of function of natural killer cells at low, and induction of apoptosis at high cereulide concentrations (Paananen et al., 2002[184]). These studies are also reflected in human cases of cereulide intoxication, in which liver (most commonly; Dierick et al., 2005[55]; Ichikawa et al., 2010[105]; Mahler et al., 1997[156]; Naranjo et al., 2011[175]; Shiota et al., 2010[209]; Tschiedel et al., 2015[220]), intestine (Naranjo et al., 2011[175]), pancreas (Pósfay-Barbe et al., 2008[191]; Thery et al., 2022[218]), kidney (Naranjo et al., 2011[175]; Pósfay-Barbe et al., 2008[191]) and/or CNS (Ichikawa et al., 2010[105]; Mahler et al., 1997[156]; Pósfay-Barbe et al., 2008[191]; Shiota et al., 2010[209]) were affected.

## Conclusion

Due to its presence in soil, *B. cereus* can easily enter the food chain, and, given the high resistance of its spores, cannot be eliminated from foodstuffs entirely. As a result, it is particularly important to gain a comprehensive knowledge of the process of toxin production and the mode of action of these toxins, irrespective of whether this involves diarrhoea-provoking enterotoxins or the emetic toxin cereulide. Although several of these processes have already been well elucidated, there are still numerous unanswered questions, e.g. regarding the highly complex interaction of various transcriptional and post-transcriptional processes in toxin gene expression, the susceptibility and response of target cells to the toxins, the specific receptor for Nhe, the expression of cereulide and its different variants, or their contribution to the overall toxicity of an isolate. It is also important to consider other secreted toxins and virulence factors such as cell wall peptidase EntFM, cereolysin O, haemolysin II, metalloproteases, collagenase or sphingomyelinase, which might interact synergistically in particular with the enterotoxins. Research outcomes and associated new and improved detection methods will continue to be implemented in routine diagnostics of food and clinical samples, leading to increasingly rapid detection of *B. cereus* food infections and intoxications and eventually to their reduction when foodstuffs are handled correctly according to HACCP standards.

## Declaration

### Conflict of interest

The authors declare that they have no conflict of interest.

### Artificial Intelligence (AI) - assisted technology

The authors declare that no type of generative artificial intelligence has been used for the writing of this manuscript, nor for the creation of images, graphics, tables, or their corresponding captions.

### Funding

This work was funded by the Deutsche Forschungsgemeinschaft (DFG, German Research Foundation) - project number 407925087.

## Figures and Tables

**Figure 1:**
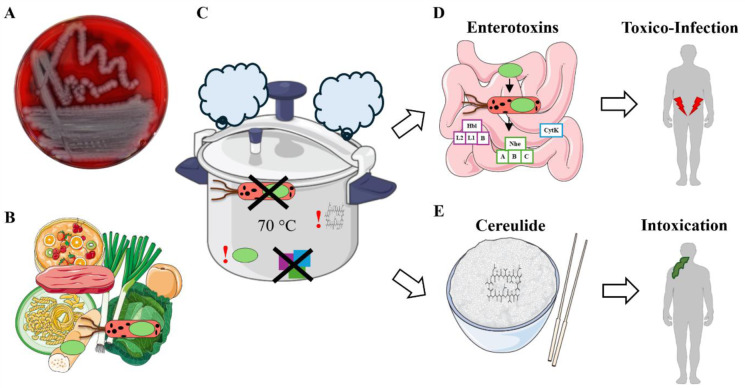
Graphical abstract: (A) Typical wax-like colony morphology of *Bacillus cereus *with haemolysis zone on sheep blood agar. (B) Contamination of different foods with vegetative bacteria (red) and/or spores (green) of this ubiquitous soil bacterium. (C) Food processing, e.g. heating usually kills/inactivates vegetative bacteria and enterotoxins, but not spores or cereulide. (D) Germination of consumed spores and enterotoxin production of vegetative cells in the intestine leads to pore formation into epithelial cells and thus, diarrhoea and abdominal pain after approx. eight to 16 hours. This process is summarised as toxico-infection. (E) Uptake of cereulide pre-formed in foods leads to nausea and vomiting, from 30 minutes to six hours after consumption. This is typical for food intoxications. Images: N. Jessberger or provided by Servier Medical Art (https://smart.servier.com/), licensed under CC BY 4.0 (https://creativecommons.org/licenses/by/4.0/)
